# Health‐related quality of life in asthma measured by the World Health Organization brief questionnaire (WHO‐BREF) and the effect of concomitant allergic rhinitis—A population‐based study

**DOI:** 10.1111/crj.13608

**Published:** 2023-04-04

**Authors:** Obianuju B. Ozoh, Sunday A. Aderibigbe, Adaeze C. Ayuk, Sandra K. Dede, Eruke Egbagbe, Musa Babashani

**Affiliations:** ^1^ Department of Medicine, College of Medicine University of Lagos Lagos Nigeria; ^2^ Department of Medicine Lagos University Teaching Hospital Lagos Nigeria; ^3^ Department of Epidemiology and Community Health University of Ilorin Ilorin Nigeria; ^4^ Department of Paediatrics, College of Medicine University of Nigeria Nsukka‐Enugu Campus Nsukka Nigeria; ^5^ Department of Paediatrics University of Nigeria Teaching Hospital Ituku/Ozalla Enugu Nigeria; ^6^ Department of Medicine University of Benin School of Medicine Benin City Edo State Nigeria; ^7^ Department of Medicine Kano Aminu Kano Teaching Hospital Kano Nigeria

**Keywords:** allergic rhinitis, asthma, quality of life, WHO‐BREF

## Abstract

**Background and objective:**

The impact of allergic rhinitis (AR), a common comorbidity in asthma, on global quality of life (QoL) using generic QoL questionnaires has not been extensively evaluated.

**Methods:**

This was a cross‐sectional population‐based study among adults ≥18 years old. Generic QoL was measured using the World Health Organization (WHO) questionnaire (WHOQOL‐BREF), and asthma control was assessed using the Asthma Control Test. Participants were categorized into four groups: Group 1 (No asthma, no AR), Group 2 (Asthma only), Group 3 (AR only) and Group 4 (Concomitant asthma and AR). The student *t*‐test or the ANOVA was used for comparison between groups and based on the level of asthma control. Linear regression was used to assess the association between the level of asthma control and QoL scores, adjusted for age and sex. A *p*‐value of less than 0.05 was considered significant for all associations.

**Results:**

There were 9115 participants; 906 (9.9%) had asthma, and 1998 (21.9%) had AR. The lowest QoL scores were in the environment domain. Mean QoL scores were significantly lower in asthma compared to ‘no asthma’ and in AR compared to ‘no AR’. Either asthma or rhinitis (Group 2 or 3) had significantly lower scores compared to no disease (Group 1) only in the environment domain, but the concomitant disease (Group 4) had lower scores across all categories and domains. Scores were significantly lower for uncontrolled asthma compared to controlled asthma and for ‘concomitant asthma and AR’ compared to ‘asthma only’. Increasing age and uncontrolled asthma predicted worse health‐related quality of life (HRQoL) consistently.

**Conclusion:**

Although asthma and AR negatively impact HRQoL independently, concomitant asthma and AR are worse. Uncontrolled asthma underpins poor QoL in asthma because QoL is not impaired in controlled disease. This underscores the need for recognition and treatment of AR in asthma and reinforces the benefits of achieving asthma control as a priority in asthma treatment.

## Graphical abstract

Uncontrolled asthma affects health‐related quality of life, but the impact of concomitant allergic rhinitis and controlled asthma on global quality of life is highlighted. The presence of asthma negatively impacts all aspects of global quality of life and predicts lower quality of life with worse outcomes with concomitant rhinitis.

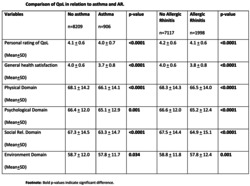

**Abbreviations**
ACT: asthma control test
AIRNIG: asthma insight and reality study in Nigeria
AR: allergic rhinitis
GINA: global initiative on asthma
HRQoL: health‐related quality of life
QoL: quality of Life

## 1 INTRODUCTION

Quality of life (QoL) is an important health metric that is independent of disease status but could be affected by the presence of the disease, the severity of the disease and the level at which the disease is controlled.^1^ It is now recognized that disease burden transcends the manifestation of symptoms and encompasses perceived QoL, which also bears on morbidity and mortality. Consideration of QoL, therefore, informs treatment decisions and enhances the delivery of holistic care.

The World Health Organization (WHO) describes QoL as the personal perception of position in life in the context of the existing culture and value systems and in relation to individual goals, expectations, standards and concerns.^2^ Health‐related quality of life (HRQoL), in contrast, is defined as ‘the value assigned to the duration of life as modified by the impairments, functional states, perceptions and social opportunities that are influenced by disease, injury, treatment or policy’.^3^

HRQoL in asthma remains essential in the assessment of overall disease burden and in the setting of treatment goals.^4^ Most studies have assessed HRQoL in asthma using disease‐specific tools such as the St George's Respiratory Questionnaire (SGRQ) and the Asthma Quality of Life Questionnaire (AQLQ), and these bear heavily on the effect of symptoms.^5^, ^6^ This is inadequate as it fails to consider other aspects of QoL, such as the presence of comorbidities and social functioning, which also substantially affect QoL and disease outcomes.^7^, ^8^

Conversely, generic QoL instruments are designed for applicability across a wide range of diseases, interventions and populations. The advantage of using these over disease‐specific questionnaires is that they provide more comprehensive information that transcends the impact of the physical symptoms of the disease to include the social and emotional dimensions. Although disease‐specific questionnaires may be more sensitive in detecting changes in clinical parameters over time, there is no difference for cross‐sectional assessments.^9^ However, the generic questionnaires have an edge because they can also be used for comparison of QoL among persons with and without diseases, with the additional benefit of a greater sensitivity to the presence of comorbidities.^1^, ^10^

Only a few studies have reported lower generic QoL in asthma associated with poorly controlled asthma using generic questionnaires such as the SF‐36 and EQ‐5D. We are unaware of the use of the World Health Organization Quality of Life Brief Version (WHO‐BREF) to assess QoL in asthma. The WHO‐BREF is a very reliable generic QoL questionnaire across different populations, countries, and cultures and is sensitive to changes in physical and social circumstances.^11^ Furthermore, the impact of allergic rhinitis (AR), a common comorbidity in asthma with immunological, epidemiological and clinical linkage, on QoL has not been extensively evaluated, particularly in the context of a general African population.^12^, ^13^

We set out to use the WHO‐BREF HRQoL questionnaire to assess the generic QoL among persons with asthma and explored the effect of the level of asthma control and the presence of concomitant asthma and AR. The generic QoL provides a holistic view of persons with asthma beyond the effects of the clinical features of the disease. It reflects how asthma may also influence social and emotional factors and overall satisfaction in life. Although it is known that concomitant AR worsens clinical asthma outcomes, a negative impact on QoL deepens the burden and further underpins its recognition.

## 2 METHODS

### 2.1 Study designs and populations

This was secondary data analysis from a cross‐sectional population study conducted between 2017 and 2018 across five major cities representing five geo‐political zones of Nigeria to assess the prevalence of asthma and the level of asthma control and state of asthma management (asthma insight and reality study in Nigeria [AIRNIG]).^14^, ^15^ The method of participant recruitment and data collection has been described previously.^15^ Validated asthma screening questionnaires^15^ were used to identify persons with asthma in the community from selected households in a stratified random manner. Clinical asthma was defined as physician‐diagnosed asthma and/or affirmation of the presence of asthma symptoms or asthma attack and the use of asthma medications within the preceding 12 months. AR was assessed using the Score for Allergic Rhinitis (SFAR) questionnaire, a validated 8‐item questionnaire that obtains information on nasal symptoms, periodicity (perennial vs. seasonal), associated conjunctivitis, specific triggers, allergic status, results of allergy test, allergy diagnosis and family history of atopy.^16^ A score of >7 (maximum score 16) on the SFAR questionnaire was considered positive for AR. For this QoL analysis, only adults 18 years old and above were included.

### 2.2 Assessment of asthma control

We assessed asthma control using two validated asthma control measures, Asthma Control Test (ACT) and the Global Initiative for Asthma (GINA) questionnaire, respectively. The adult version of the ACT is a 5‐item questionnaire that assesses asthma symptoms and the use of asthma medications in the preceding 4 weeks. Scores on the ACT range from 0 to 25, and scores of 19 and below are categorized as ‘uncontrolled’, whereas >19 denotes ‘controlled’ asthma.^16^ The dichotomized 4‐point GINA questionnaire assesses daytime symptoms more than twice a week, any night‐time symptoms, reliever needed for symptoms more than twice a week and any limitation in activity due to asthma; and categorizes the level of control as ‘well controlled’ when none of these symptoms is present, ‘partly controlled’ when one to two symptoms only are present and ‘uncontrolled’ if three to four of these symptoms are present.^17^ The two questionnaires were used to increase the sensitivity and comparability of our findings with other studies.

### 2.3 Measurement of generic QoL

We assessed generic QoL using the WHO QoL questionnaire, the WHOQOL‐BREF. This questionnaire is recommended for use in a variety of cultural settings, the presence or absence of disease, and is validated for different populations, including Nigerians.^2^ WHOQOL‐BREF comprises 26 questions that address individual perceptions of health and well‐being over the preceding 2 weeks. Responses to the questions on the WHOQOL‐BREF are on a 1–5 Likert scale where 1 represents ‘disagree’ or ‘not at all’ and 5 represents ‘completely agree’ or ‘extremely’. All scores are scaled in a positive direction (i.e., higher scores denote higher QoL).

The first two questions are categorized into two separately scored items:

1. *Item 1:* ‘How would you rate your QoL?’
2. *Item 2:* ‘What is your general satisfaction with your health?’

The other 24 questions cover four domains, each with specific facets as follows:

1. *Domain 1*: Physical health (activities of daily living, dependence on medicinal substances and medical aids, energy and fatigue, mobility, pain and discomfort, sleep and rest and work capacity).
2. *Domain 2:* Psychological health (bodily image and appearance, negative feelings, positive feelings, self‐esteem, spirituality/religion/personal beliefs and thinking, learning, memory and concentration).
3. *Domain 3:* Social relationships (personal relationships, social support and sexual activity).
4. *Domain 4:* Environment (financial resources, freedom, physical safety and security, health and social care: accessibility and quality, home environment, opportunities for acquiring new information and skills, participation in and opportunities for recreation/leisure activities and physical environment, (pollution/noise/traffic/climate).

### 2.4 Ethical considerations

Ethical approval was obtained from the Health Research Ethics Committees of all participating institutions and from the National Health Research Ethics Committee of the Federal Ministry of Health, Nigeria. Informed consent was obtained from the head of each household and from individual participants.

### 2.5 Statistical analyses

We transformed the four domain scores to range from 0 to 100 using the standard scoring instructions for using the WHO‐BREF. The mean of the two stand‐alone questions and the four domains, respectively, were compared based on having asthma and AR, respectively, using the student *t*‐test. The participants were also categorized into four mutually exclusive groups: Group 1 (no asthma, no AR), Group 2 (asthma only), Group 3 (AR only), Group 4 (concomitant asthma and AR), and the mean values compared using the student *t*‐test. The mean QoL scores were compared based on the level of asthma control using the ACT and the GINA questionnaires, respectively, among participants with asthma, using the student *t*‐test and the analysis of variance (ANOVA) as appropriate, with a post hoc Tukey analysis. Linear regression models were used to determine the association between the level of asthma control and QoL adjusted for age and sex. A *p*‐value of less than 0.05 was considered significant for all associations.

Minimal clinical important difference (MCID) is the smallest change in QoL score that a person considers important and clinically relevant. For the WHO‐BREF, MCID has not been widely reported, but a study among patients with early breast cancer using the WHO‐100 questionnaire found one point to be the MCID. Because we are using transformed scores for analysis in this study, we considered a one‐point difference between group means scores as the MCID.^18^

## 3 RESULTS

### 3.1 Participant characteristics

A total of 9115 adults completed the WHOQOL‐BREF questionnaire. Of these, 906 (9.9%) had asthma and 1998 (21.9%) had AR. Distribution across the four mutually exclusive groups were as follows: Group 1 (no asthma, no AR) 6839 (75%); Group 2 (asthma only) 278 (3%); Group 3 (AR only) 1370 (15%); Group 4 (concomitant asthma and AR) 628 (7%).

Table 1 describes the socio‐demographic characteristics and QoL scores of the participants. Over half of the participants earned less than $50 monthly. The lowest QoL scores were in the environment domain for all study participants. Males had lower QoL scores in the physical health and social relationship domains compared to females, which were MCID.

**TABLE 1:** Socio‐demographic characteristics of participants and QoL scores.

Variables	All study participants *N* = 9115 (%)	Male *n* = 3855 (%)	Female *n* = 5260 (%)	*p*‐value
Age (years): Median (IQR)	34.0 (19)	35.0 (20)	34.0 (19)	
Highest level of education				<0.0001
None	542 (5.9)	74 (1.9)	468 (8.9)	
Primary	1899 (20.8)	687 (17.8)	1212 (23.0)	
Secondary	5606 (61.5)	2505 (65.0)	3101 (59.0)	
University graduate	1068 (11.7)	589 (15.3)	479 (9.1)	
Household income (Naira)				<0.0001
<18 000	3524 (38.7)	1139 (29.5)	2385 (45.3)	
18 000–35 000	1818 (19.9)	823 (21.3)	995 (18.9)	
36 000–53 000	1960 (21.5)	988 (25.6)	972 (18.5)	
54 000–71 000	986 (10.8)	499 (12.9)	487 (9.3)	
72 000–89 000	448 (4.9)	205 (5.3)	243 (4.6)	
>90 000	379 (4.2)	201 (5.2)	178 (3.4)	
Smoking status				<0.0001
Current	185 (2.0)	176 (4.6)	9 (0.2)	
Ex‐smoker	166 (1.8)	159 (4.1)	7 (0.1)	
Non‐smoker	8764 (96.2)	3520 (91.3)	5244 (99.7)	
Personal rating of QoL (Mean ± SD)	4.1 ± 0.6	4.1 ± 0.6	4.1 ± 0.6	0.136
General health satisfaction (Mean ± SD)	4.0 ± 0.7	4.0 ± 0.7	4.0 ± 0.6	0.104
Physical health domain (Mean ± SD)	67.9 ± 14.2	66.8 ± 14.1	68.7 ± 14.3	<0.0001*
Psychological health domain (Mean ± SD)	66.3 ± 12.1	66.7 ± 12.0	66.0 ± 12.2	0.005
Social relationships domain (Mean ± SD)	66.9 ± 14.6	65.8 ± 14.9	67.7 ± 14.3	<0.0001*
Environment domain (Mean ± SD)	58.6 ± 11.9	59.2 ± 12.1	58.2 ± 11.8	<0.0001*

*Note*: Bold *p*‐values indicate significant difference between males and females.

Abbreviations: IQR, interquartile range; QoL, quality of life; SD, standard deviation.

* Minimal clinical important difference (MCID).

### 3.2 Comparison of QoL in relation to asthma and AR

Table 2 shows the comparison of QoL scores between participants in relation to having asthma and AR, respectively. QoL scores were significantly lower in participants with asthma compared to those with ‘no asthma’ across both stand‐alone items and all the domains with MCID in three domains. QoL scores were also significantly lower for AR compared to ‘no AR’ with MCID across all domains.

**TABLE 2:** Comparison of QoL in relation to asthma and AR.

Variables	No asthma *n* = 8209	Asthma *n* = 906	*p*‐value	No allergic rhinitis *n* = 7117	Allergic rhinitis *n* = 1998	*p*‐value
Personal rating of QoL (Mean ± SD)	4.1 ± 0.6	4.0 ± 0.7	<0.0001	4.2 ± 0.6	4.1 ± 0.6	<0.0001
General health satisfaction (Mean ± SD)	4.0 ± 0.6	3.7 ± 0.8	<0.0001	4.0 ± 0.6	3.8 ± 0.8	<0.0001*
Physical domain (Mean ± SD)	68.1 ± 14.2	66.1 ± 14.1	<0.0001*	68.3 ± 14.3	66.5 ± 14.0	<0.0001*
Psychological domain (Mean ± SD)	66.4 ± 12.0	65.1 ± 12.9	0.001*	66.6 ± 12.0	65.2 ± 12.4	<0.0001*
Social rel. domain (Mean ± SD)	67.3 ± 14.5	63.3 ± 14.7	<0.0001*	67.5 ± 14.4	64.9 ± 15.1	<0.0001*
Environment domain (Mean ± SD)	58.7 ± 12.0	57.8 ± 11.7	0.034*	58.8 ± 11.8	57.8 ± 12.4	0.001*

*Note*: Bold *p*‐values indicate significant difference.

Abbreviations: AR, allergic rhinitis; QoL, quality of life; SD, standard deviation.

* Minimal clinical important difference (MCID).

Table 3 shows the comparison of QoL scores between Group 1 (no asthma, no AR) and the other groups, respectively. ‘Asthma only’ had significantly lower scores with MCID only in the social relationship domain compared to ‘no asthma, no AR’. ‘AR only’ also had a significantly lower score with MCID in the social relationship domain compared to ‘no asthma, no AR’. ‘Concomitant asthma and AR’ had significantly lower scores across all items and domains compared to ‘no asthma, no AR’ but without MCID in the general health satisfaction domain.

**TABLE 3:** Comparison of HRQoL between ‘no asthma, no AR’ with the ‘asthma only’, ‘AR only’ and ‘concomitant asthma and AR’, respectively.

QoL scores	Group 1 (no asthma, no AR) *n* = 6839	Group 2 (asthma only) *n* = 278	*p*‐value	Group 3 (AR only) *n* = 1370	*p*‐value	Group 4 (concomitant asthma and AR) *n* = 628	*p*‐value
Personal rating of QoL (Mean ± SD)	4.1 ± 0.6	4.2 ± 0.7	0.190	4.1 ± 0.6	0.252	3.9 ± 0.6	<0.0001*
General health satisfaction (Mean ± SD)	4.0 ± 0.6	4.0 ± 0.7	0.389	4.0 ± 0.7	<0.0001	3.5 ± 0.9	<0.0001
Physical health domain (Mean ± SD)	68.2 ± 14.2	70.6 ± 14.4	0.005*	67.6 ± 14.2	0.147	64.1 ± 13.4	<0.0001*
Psychological health domain (Mean ± SD)	66.5 ± 11.9	69.4 ± 12.3	0.0001*	66.1 ± 12.2	0.346	63.1 ± 12.7	<0.0001*
Social relationship domain (Mean ± SD)	67.7 ± 14.3	62.7 ± 15.8	<0.0001*	65.5 ± 15.5	<0.0001*	63.5 ± 14.2	<0.0001*
Environment domain (Mean ± SD)	58.8 ± 11.8	59.2 ± 12.5	0.610	58.1 ± 12.9	0.057	57.2 ± 11.3	0.001*

*Note*: Group 2, 3 and 4, respectively, were compared to group 1. Bold *p*‐values indicate statistically significant differences. Comparisons between groups ‘AR only’ and ‘asthma only’ showed that ‘AR only’ had statistically significantly lower scores with MCID in the physical domain (*p* = 0.001) and psychological domain (*p* < 0.001) but higher scores in the social relations domain (*p* = 0.006).

Abbreviations: AR, allergic rhinitis; HRQoL, health‐related quality of life; QoL, quality of life; SD, standard deviation.

* Minimal clinical important difference (MCID).

### 3.3 Effect of concomitant rhinitis and asthma control on QoL in asthma

Table 4 compares QoL scores between ‘asthma only’ and ‘concomitant asthma and AR’.

**TABLE 4:** Comparison of Qol score between ‘asthma only’ and ‘concomitant asthma and AR’.

QoL domains	Asthma only (*n* = 278)	‘Concomitant asthma and AR’ (*n* = 628)	*p*‐value
Overall quality of life (Mean ± SD)	4.2 ± 0.7	3.9 ± 0.6	<0.001
General health satisfaction (Mean ± SD)	4.0 ± 0.7	3.5 ± 0.9	<0.001
Physical domain (Mean ± SD)	70.6 ± 14.4	64.1 ± 13.4	<0.001*
Psychological domain (Mean ± SD)	69.4 ± 12.3	63.1 ± 12.7	<0.001*
Social rel. domain (Mean ± SD)	62.7 ± 15.8	63.5 ± 14.2	0.42
Environment domain (Mean ± SD)	59.2 ± 12.5	57.2 ± 11.3	0.02*

*Note*: Bold *p*‐values indicate statistically significant differences.

Abbreviations: AR, allergic rhinitis; QoL, quality of life; SD, standard deviation.

* Minimal clinical important difference (MCID).

Participants with ‘Concomitant asthma and AR’ had significantly lower scores with MCID in the physical, psychological and environmental domains.

Table 5 compares QoL scores based on the level of asthma control using the ACT score and the GINA score, respectively. QoL was significantly lower with MCID across the physical, psychological and environmental domains among participants with uncontrolled asthma compared to those with controlled asthma using the ACT. This was similar using the GINA assessment tools. The scores between partly controlled asthma and controlled asthma in the GINA category were similar.

**TABLE 5:** Comparison of mean QoL scores based on level of asthma control.

QoL scores	Level of asthma control (ACT)			Level of asthma control (GINA)			
	Controlled asthma (*n* = 388)	Uncontrolled asthma (*n* = 360)	*p*‐value	Well controlled asthma (*n* = 46)	Partly controlled asthma (*n* = 548)	Uncontrolled asthma (*n* = 154)	*p*‐value
Personal rating of quality of life (Mean ± SD)	4.1 ± 0.6	3.8 ± 0.8	<0.0001*	4.0 ± 0.5^a^	4.1 ± 0.6^b^	3.6 ± 0.8^c^	<0.0001
General health satisfaction (Mean ± SD)	3.7 ± 0.7	3.4 ± 1.0	<0.0001	3.8 ± 0.8^a^	3.7 ± 0.8^b^	3.1 ± 0.9^c^	<0.0001
Physical domain (Mean ± SD)	67.1 ± 13.1	61.5 ± 14.1	<0.0001*	63.1 ± 11.2^a^	66.7 ± 13.6^b^	56.6 ± 12.8^c^	<0.0001*
Psychological domain (Mean ± SD)	66.9 ± 11.5	60.8 ± 13.4	<0.0001*	67.0 ± 13.8^a^	65.7 ± 11.6^b^	56.9 ± 14.3^c^	<0.0001*
Social relationship domain (Mean ± SD)	64.2 ± 14.0	61.9 ± 15.7	0.037*	66.4 ± 11.4	63.0 ± 15.1	62.5 ± 14.7	0.274
Environment domain (Mean ± SD	60.0 ± 10.6	54.8 ± 12.3	<0.0001*	63.5 ± 9.2^a^	58.3 ± 11.4^b^	52.9 ± 12.1^c^	<0.0001*

*Note*: Asthma control measures were available for 748 participants. Bold *p*‐values indicate significant difference between levels of asthma control. Tukey Post‐Hoc Test; Personal rating of QoL (a vs. b, *p* = 0.948; a vs. c, *p* < 0.0001; b vs. c, *p* < 0.0001): General Health Satisfaction (a vs. b, *p* = 0.799; a vs. c, *p* < 0.0001; b vs. c, *p* < 0.0001): Physical domain (a vs. b, *p* = 0.162; a vs. c, *p* = 0.013; b vs. c, *p* < 0.0001): Psychological domain (a vs. b, *p* = 0.754; a vs. c, *p* < 0.0001; b vs. c, *p* < 0.0001); Environment domain (a vs. b, *p* = 0.009; a vs. c, *p* < 0.0001; b vs. c, *p* < 0.0001).

Abbreviations: ACT, Asthma Control Test; GINA, Global Initiative on Asthma.

* Minimal clinical important difference (MCID).

When compared to those without asthma (Table S1), those with uncontrolled asthma had significantly lower scores across all items and domains.

### 3.4 Associations between asthma control and QoL

Table 6 shows the association between HRQoL and level of asthma control based on the ACT, adjusted for age and sex. Controlled asthma was associated with higher QoL scores in univariate and bivariate analyses across all categories. In the adjusted model, increasing age was associated with poorer QoL. Female sex was associated with better QoL in the personal rating of QoL, physical and social relationship domains.

**TABLE 6:** Association between asthma control and QoL.

Variables	Standardized coefficient B	95% CI	*p*‐value	Standardized coefficient B	95% CI	*p*‐value
	Personal rating of QoL					
	Unadjusted			Adjusted		
Age	−0.169	−0.011, −0.005	<0.001	−0.109	−0.008, −0.002	0.003
Sex (female)	0.089	0.033, 0.209	0.007	0.074	0.005, 0.197	0.039
Controlled asthma	0.191	0.163, 0.355	<0.001	0.179	0.147, 0.339	<0.001
	General health satisfaction					
Age	−0.168	−0.013, −0.006	<0.001	−0.120	−0.011, −0.003	0.001
Sex (female)	0.056	−0.015, 0.207	0.091	0.051	−0.034, 0.209	0.158
Controlled asthma	0.183	0.192, 0.435	<0.001	0.169	0.169, 0.412	<0.001
	Physical domain					
Age	−0.217	−0.267, −0.146	<0.001	−0.166	−0.224, −0.092	<0.001
Sex (female)	0.084	0.551, 4.238	0.011	0.101	0.875, 4.745	0.004
Controlled asthma	0.202	3.656, 7.564	<0.001	0.184	3.176, 7.042	<0.001
	Psychological domain					
Age	−0.212	−0.242, −0.130	<0.001	−0.169	−0.209, −0.087	<0.001
Sex (female)	0.024	−1.082, 2.318	0.476	0.028	−1.049, 2.516	0.420
Controlled asthma	0.240	4.361, 7.943	<0.001	0.219	3.845, 7.406	<0.001
	Social relationship domain					
Age	−0.154	−0.217, −0.089	<0.001	−0.172	−0.247, −0.103	<0.001
Sex (female)	0.116	1.510, 5.346	<0.001	0.127	1.714, 5.921	<0.001
Controlled asthma	0.076	0.132, 4.397	0.037	0.058	−0.370, 3.833	0.106
	Environment domain					
Age	−0.156	−0.175, −0.073	<0.001	−0.130	−0.161, −0.048	<0.001
Sex (female)	0.025	−0.939, 2.130	0.447	0.023	−1.099, 2.197	0.513
Controlled asthma	0.222	3.571, 6.861	<0.001	0.207	3.199, 6.491	<0.001

## 4 DISCUSSION

In this study, we used the generic WHO‐BREF questionnaire to assess QoL in asthma and AR to show the impact of these diseases on global QoL beyond those attributable to physical symptoms of the diseases. It also enabled us to compare QoL between persons with and without disease and to account for comorbidities such as concomitant asthma and AR. The main finding in this study is that asthma and AR negatively impact generic QoL measured using the WHO‐BREF questionnaire, but concomitant asthma and AR have a worse impact. Furthermore, QOL is worse in uncontrolled asthma than controlled asthma, and persons with controlled asthma had similar QoL compared to those without asthma. We note that although QoL was significantly lower among persons with asthma compared to “no asthma”, sensitivity analysis based on the four mutually exclusive groups showed that asthma only, without rhinitis, has similar QoL scores compared to neither asthma nor AR.

AR is a frequently undertreated condition that is often considered innocuous, but we show and support previous reports that although it may not be life‐threatening, it adversely impacts QoL.^19^ AR is a common comorbidity in asthma, occurring in up to 70% of persons with asthma, and associated with an increase in asthma morbidity when left untreated.^14^, ^15^ Our findings extend the dimensions of the negative impact of AR on asthma beyond the clinical impact on disease morbidity and underscore the need to identify and treat AR in asthma. Similar to our findings, Jansson et al.^13^ have also reported that HRQoL is worse among persons with asthma who have concomitant AR compared to those with either disease. The negative impact of AR on QoL may stem from the high symptom burden, including runny nose, mouth breathing, change in phonation, daytime somnolence and poor academic performance, which could lead to stigma.^20^ Therefore, recognition and treatment of concomitant AR in asthma is important for the reduction of the negative effect on asthma control and also to mitigate the negative impact on QoL.^20^, ^21^, ^22^

A few studies have assessed the impact of asthma on generic QoL, which provides an all‐inclusive assessment beyond the physical impact of the disease.^12^, ^13^ In a study using the generic SF‐12 questionnaire, worse QOL was also reported, but it was limited to the physical component. Both the SF‐12 and the WHO‐BREF questionnaires are reliable for assessing generic QoL in different populations.^23^, ^24^ However, the WHO‐BREF has been found to measure the global QoL more reliably due to its assessment of factors beyond just physical attributes.^23^, ^25^, ^26^ Our finding of worse QoL in the social and psychological domains among persons with asthma using the WHO‐BREF may be attributed to adverse social and economic factors, including high out‐of‐pocket costs experienced by persons with asthma in the country which are made more profound by the low socioeconomic status of most of the participants in our study.^14^, ^27^, ^28^

QoL scores were comparable between persons without asthma and those with controlled asthma, similar to previous reports.^13^ It demonstrates the clear benefits of optimal asthma management to improve asthma control. Controlled asthma is achievable and hinges on patient education and adherence to guideline‐recommended treatment. Access to treatment for all asthma patients, especially in low‐resource settings, should be optimized, and assessment of QoL using generic questionnaires made an important outcome measure that guides intervention.

The environment domain scores, which assess financial resources and the opportunities it provides, were the lowest for all participants in this study. With over 50% of all the participants earning the equivalent of less than $50 monthly, the achievement of the metrics in this domain is unlikely. Data from the World Bank demonstrates very high rates of poverty in Nigeria, with over 60% of the population living in poverty.^29^ Notable is that participants with controlled asthma had better environment domain scores compared to those without asthma. It may be related to the influence of self‐care measures taken to control asthma that could include improvement in the physical environment. Environmental factors are central to the clinical manifestations of asthma and the level of asthma control.^30^

We also found that older age consistently influenced QoL negatively. The higher frequency of comorbidities with older age and poor social support for the elderly in Nigeria may contribute to this.^31^ The effect of sex was nuanced, but being female predicted better HRQoL in the personal rating of QoL, physical domain and social domains. The patriarchal African society with a greater burden of family responsibilities attributed to men may contribute to their experience of poorer QoL.^32^, ^33^

The large sample size selected from a general population and the use of two methods for assessing asthma control in this study are important strengths that improve the comparability of our findings with previous studies. We also note that the study may have been overpowered, and this may account for significant differences that may not be clinically meaningful. Identifying differences that were of MCID enabled us to identify those that are more likely to be relevant. We note that the body mass index, which may influence QoL, was not included in the regression model, but the inclusion of the presence of comorbidities did not influence the predictors.^34^

## 5 CONCLUSION

We have shown that the impact of asthma and AR on QoL transcend the effect of symptoms by using a generic questionnaire in this study. Although asthma and AR negatively impact HRQoL independently, concomitant asthma and AR have a worse impact. Poor QoL in asthma is driven by uncontrolled disease, as persons with controlled asthma have similar QoL as those without asthma. This underscores the need for recognition and treatment of AR in asthma and reinforces the benefits of achieving asthma control as a priority in asthma treatment.

## AUTHOR CONTRIBUTIONS

**Obianuju B. Ozoh**: Conceptualization; methodology; data curation; writing—original draft preparation; visualization; investigation supervision; validation reviewing and editing. **Sunday A. Aderibigbe**: Software; data curation visualization; investigation reviewing and editing. **Adaeze C. Ayuk**: Conceptualization; methodology; writing—original draft preparation; investigation supervision; validation reviewing and editing. **Sandra K. Dede**: Investigation supervision; validation reviewing and editing. **Eruke Egbagbe**: Investigation supervision; validation reviewing and editing. **Musa Babashani**: Investigation supervision; validation reviewing and editing. All authors approved the final draft for submission.

## CONFLICT OF INTEREST STATEMENT

None declared.

## ETHICS STATEMENT

Ethical approval was obtained from the ethical review committees of all participating institutions and from the National Health Research Ethics Committee of the Federal Ministry of Health of Nigeria.

## Supporting information


**Table S1:** Comparison with “No asthma” based on level of asthma control

## Data Availability

The data that support the findings of this study are available from the corresponding author, [ACA], upon reasonable request.
