# Supplementary material for: Health‐related quality of life in asthma measured by the World Health Organization brief questionnaire (WHO‐BREF) and the effect of concomitant allergic rhinitis—A population‐based study
Source: Clin Respir J. 2023 Apr 4;19(3):e13608. doi: 10.1111/crj.13608 (PMC11931326; doi:10.1111/crj.13608)
Supplement: Supplementary file 1 — Table S1: Comparison with “No asthma” based on level of asthma control [file CRJ-19-e13608-s001.docx]

**Table S1: Comparison with “No asthma” based on level of asthma control**

| **QoL scores** | **No Asthma**  **n=8209** | **Controlled asthma (ACT)**  **n=388** | **p-value** | **Uncontrolled asthma (ACT)**  **n=360** | **p-value** | **Well Controlled (GINA) n=46** | **p-value** | **Partly controlled (GINA)**  **n=548** | **p-value** | **Uncontrolled n=154** | **p-value** |
| --- | --- | --- | --- | --- | --- | --- | --- | --- | --- | --- | --- |
| **Personal rating of quality of life (Mean+SD)** | 4.2 + 0.6 | 4.1 + 0.6 | 0.175 | 3.8 + 0.8 | **<0.0001** | 4.0 + 0.5 | 0.253 | 4.1 + 0.6 | **0.008** | 3.6 + 0.8 | **<0.0001** |
| **General health satisfaction (Mean+SD)** | 4.0 + 0.6 | 3.7 + 0.7 | **<0.0001** | 3.4 + 1.0 | **<0.0001** | 3.8 + 0.7 | **0.013** | 3.7 + 0.8 | **<0.0001** | 3.1 + 0.9 | **<0.0001** |
| **Physical Domain (Mean+SD)** | 68.1 + 14.2 | 67.1 + 13.1 | 0.172 | 61.5 + 14.1 | **<0.0001*** | 63.0 + 11.2 | **0.015*** | 66.7 + 13.6 | **0.027*** | 56.6 + 12.8 | **<0.0001*** |
| **Psychological Domain (Mean+SD)** | 66.4 + 12.0 | 66.9 + 11.5 | 0.396 | 60.8 + 13.4 | **<0.0001*** | 67.1 + 13.6 | 0.713 | 65.7 + 11.6 | 0.184 | 56.9 + 14.3 | **<0.0001*** |
| **Social Relationship Domain (Mean+SD)** | 67.3 + 14.5 | 64.2 + 14.0 | **<0.0001*** | 61.9 + 15.7 | **<0.0001*** | 66.6 + 11.3 | 0.755 | 63.0 + 15.1 | **<0.0001*** | 62.5 + 14.7 | **<0.0001*** |
| **Environment Domain (Mean+SD)** | 58.7 + 12.0 | 60.0 + 10.6 | **0.031*** | 54.8 + 12.3 | **<0.0001*** | 63.5 + 9.1 | **0.007*** | 58.3 + 11.4 | 0.459 | 52.9 + 12.1 | **<0.0001*** |

Footnote: asthma control measures were available for 748 participants. Bold p-value indicate significant association SD=Standard deviation. *Minimal Clinical Important Difference (MCID)
